# On the (relation between) efficiency and secret key rate of QKD

**DOI:** 10.1038/s41598-024-54246-y

**Published:** 2024-02-13

**Authors:** Georgi Bebrov

**Affiliations:** Telecommunications Department, Technical University of Varna, 1 Studentska Street, 9010 Varna, Bulgaria

**Keywords:** Quantum key distribution, Key rate, Efficiency, Quantum information, Qubits

## Abstract

The processes of evaluation and comparison play a vital role in the development of a scientific field. In the field of quantum cryptography (especially quantum key distribution, QKD), the so-called secret key rate is used for characterizing the performance of a protocol (scheme). However the current definition of this quantity is incomplete. It does not consider the classical communication process taking place in a QKD protocol. There exists a quantity that involves all the procedures (resources) in a communication process: it is the efficiency (total efficiency). This paper reports a definition of this parameter. Also the relation between the total efficiency and key rate is found. By means of this relation, the total secret key rate of a QKD protocol is expressed. An application of the total key rate is demonstrated: the original twin-field QKD (TF-QKD) is evaluated in terms of this rate. The paper also shows a comparison between the total key rate and the standard key rate of a TF-QKD.

## Introduction

The quantum mechanics, when applied to the problem of establishing secrets between remote parties, provides incredible results, both in theory and in practice. The product of this act is the so-called quantum key distribution (QKD) which is initially developed by Bennett and Brassard in 1984^1^. In the subsequent years several rationalizations of the initial QKD model were proposed^2–6^. A real practical development was made by Hwang in his seminal paper^7^. There the author proposed the so-called decoy-state method which ensures both security against photon-number splitting attack and increase in the operation distance of a QKD system. Another progress in the field of QKD is the measurement-device-independent QKD scheme which achieves relatively high operation distances^8^. A masterful advance was made by Lucamarini et al., who showed that it is possible to realize a QKD scheme whose key rate scales with the square root of the transmittance, thereby displaying that further operation distances are reachable^9^. The scheme is called twin-field quantum key distribution (TF-QKD).

The parameter *secret key rate* (more precisely the function of the secret key rate in terms of operation distance) appears to characterize almost entirely the QKD systems^9,10^. Another characterizing parameter, which could be regarded as a complement of the key rate, is the *efficiency*^11^. While the way of determining the key rate of distinct QKD protocols is well-known (a general expression for the key rate exists), there is no general equation for determining the efficiency (total efficiency) of a QKD system. An attempt for developing an efficiency expression is made in Ref.^12^. However, it seems to be inadequate, because of its incompleteness. The incompleteness follows from the fact that the efficiency proposed in^12^ is not capable of taking the value of zero, which corresponds to either high error rate or finding the presence of an eavesdropper in a QKD session (one run of the QKD protocol). Another problem in the field of QKD is the lack of correspondence between key rate and efficiency, which (by definition) share a lot in common.

In the present paper definitions (expressions) of both qubit efficiency and total efficiency are introduced. Based on these expressions, the relation between efficiency and key rate is clearly brought out. A revision to the notion of secret key rate is put forward. A quantity, named *total key rate* is presented. It is defined as a product of the total efficiency and the pulse repetition frequency of the source (combination of clock rates of both quantum and classical sources). According to its definition, the total key rate could be assumed to describe a QKD system in a complete manner.

The paper is organized as follows. Section “Key rate” recalls the standard definition of a secret key rate. Also, definitions for communication rate and communication process are introduced. Section “Relation between efficiency and rate” presents definitions of qubit efficiency, total efficiency, and total key rate. Moreover, the relation between efficiency and key rate is put forward. Section “Results” displays results concerning the total key rate of a twin-field quantum key distribution protocol. Comparison is made between the newly-proposed total key rate and the standard key rate concept. Section “Discussions” puts some discussions forward. The discussions are concerned with: (1) applying the efficiency evaluation to quantum secure communication schemes; (2) behavior of the efficiency (key rate) in terms of finite-size effects; and (3) behavior of the efficiency (key rate) when distinct key reconciliation and privacy amplification algorithms are performed. Section “Conclusion” sets out the conclusions.

## Key rate

Perhaps the first definition of a secret key rate is given by^10^. It has the form

1 $$\begin{aligned} R = 1 - H_2(\delta ) - H_2(\delta _p), \end{aligned}$$

where $H_2(\delta )$ denotes the Shannon entropy of a probabilistic variable $\delta$. Here $H_2(\delta )$ is used to represent the fraction of the sifted key bits being sacrificed to perform error correction, whereas $H_2(\delta _p)$ is used to represent the fraction of the sifted key bits being sacrificed to perform privacy amplification^10^. In Ref.^10^, the authors regard *R* as *asymptotically achievable rate of extraction of secret final key from sifted key*, in brief *key generation rate*. The variables $\delta$, $\delta _p$ reflect the quantum bit error rate (QBER) of the quantum channel. One could consider the terms $H_2(\delta )$ and $H_2(\delta _p)$ as *reduction coefficients* of the rate: (1) *R* = 1 is the maximum value of the rate; (2) both $H_2(\delta )$ and $H_2(\delta _p)$ appear with a minus sign in the expression.

There is more general and precise form of the above expression. It is

2 $$\begin{aligned} R = 1 - f\cdot H(\text {QBER}) - H(\text {QBER}), \end{aligned}$$

where *f* is the efficiency of the error correction algorithm (*f* $\ge$ 1). The rate expression could also incorporate quantities, which take into account the procedures of parameter estimation and sifting:

3 $$\begin{aligned} R = s\cdot p\cdot [1 - f\cdot H(\text {QBER}) - H(\text {QBER})], \end{aligned}$$

where *s* is a coefficient characterizing the sifting and *p* is a coefficient characterizing the parameter estimation. The coefficient *s* reflects the fraction of raw key bits, which remain after the sifting procedure. The coefficient *p* reflects the fraction of sifted key bits, which remain after the parameter estimation.

There is also another QKD procedure being commonly used. It is the so-called *decoy-state method*. As shown in Ref.^9^, a coefficient is used to identify the fraction of qubits (or key bits established out of those qubits), which remain after the decoy-state method. Those are the so-called *single-photon weak coherent pulses* (single qubits). Taking into account this procedure, the rate becomes

4 $$\begin{aligned} R = s\cdot p\cdot [d\cdot (1 - H(\text {QBER})) - f\cdot H(\text {QBER}) ]. \end{aligned}$$

Furthermore, one should also consider the standard procedure of transfer/measurement, which is accompanied with loss of qubits during their transit over the communication channel and their detection at the receiving station. For this reason, an additional coefficient is involved in the expression of the QKD rate:

5 $$\begin{aligned} R = s\cdot p\cdot [q\cdot d\cdot (1 - H(\text {QBER})) - q \cdot f\cdot H(\text {QBER})]. \end{aligned}$$

The coefficient *q* represents the fraction of qubits, which remain after the procedure transfer/measurement.

In this way, we obtain a quantity, which involves all the usual procedures of a QKD protocol: transfer/measurement (*q*), decoy-state method (*d*), sifting (*s*), parameter estimation (*p*), error correction ($f\cdot H(\text {QBER})$), and privacy amplification ($H(\text {QBER})$). A practical example of such a quantity is the rate *R* of Ref.^9^ (Eq. (2) of Ref.^9^).

Even though the quantity *R* is considered as key rate^9^, it differs from the standard definition (concept) of a rate. The most evident difference is that *R* is given in [*bits*] whereas an actual rate is given (measured) in [*bits*/*s*]. In order to obtain the rate of a QKD, one makes use of the expression, see for reference the supplementary material of^9^,

6 $$\begin{aligned} \mathcal {R} = c \cdot R, \end{aligned}$$

where *c* is the pulse repetition frequency of the source (clock rate), measured in Hz.

To be as precise as possible about the concept of a QKD rate, we present the following definitions:

### Definition 1

(*Data rate* or *Communication rate*). A measure of the transferred (and received) information per unit time in a communication process.

### Definition 2

(*Communication process*). A set of procedures related to communicating information from one point to another. The set consists of: message choice, signal preparation, encoding, transfer, decoding (detection and message extraction).

According to Definition 2, the data rate does not involve only the transfer rate, but also it involves the processing rate of the communication system. For the sake of simplicity, in this article, we assume that the processing rate of a QKD system is “infinite”, i.e., all the data and signal processing procedures are executed all at once. In other words, the procedures does not cause time delay during the QKD process. In this case, the rate of a QKD is given by Eq. (6).

## Relation between efficiency and rate

We define two efficiencies hereafter: (1) qubit efficiency; and (2) total efficiency. We also relate these quantities to the rate of a QKD protocol (process). We start off with qubit efficiency. This parameter has been already defined in Ref.^12^. However the definition put forward in^12^ is erroneous (inadequate). It does not account for the fact that the efficiency could go to zero (this happens in the case of high bit error rate). In this regard, the following definitions for the qubit efficiency are introduced:

### Definition 3

(*Qubit efficiency*). A ratio between the size of the cryptographic key *K* established by a QKD and the amount of qubit resources (qubits or weak coherent pulses, WCPs) *Q* used in a QKD.

### Definition 4

(*Qubit efficiency*). Quantity that reflects the amount of secretly established key bits per qubit unit.

Both definitions are characterized with the following equation:

7 $$\begin{aligned} E = \frac{K}{Q}. \end{aligned}$$

The quantity *K* is a function of *Q* (*K* = *f*(*Q*)). It could be represented as *K* = $r\cdot Q$, where *r* is a reduction coefficient. The coefficient takes into account all the procedures that involve discarding of bits during the QKD process. Such procedures are qubit (WCP) transfer, qubit (WCP) detection, sifting, parameter estimation (incorporating the decoy-state method), error correction (key reconciliation), and privacy amplification. The role of a reduction coefficient could be played by the quantity key rate *R*^9^ (Eqs. (2) or (3) of^9^). The parameter *R* is considered as reduction, because, as can be seen (verified) in^9^ and other references, it reflects the reduction of information content of a transferred qubit (WCP) in a QKD protocol. Verification is given in the previous section.

Substituting $R\cdot Q$ for *K* in Eq. (7), one obtains

8 $$\begin{aligned} E = \frac{K}{Q} = \frac{R \cdot Q}{Q} = R. \end{aligned}$$

The identity $E=R$ holds if and only if *R* takes into account all the procedures in a QKD protocol. This formula shows that the *qubit efficiency* is identical to the so-called *key rate*. This result implies that *E* and *R* are interchangeable. That is to say, we have $\mathcal {R}$ = *c* $\cdot$ *E* by utilizing both Eqs. (6) and (8).

Now let us define the *total efficiency*. Its definition is the following one:

### Definition 5

(*Total efficiency*). Quantity that reflects the amount of secretly established key bits per resource unit.

A *resource unit * (for the sake of brevity, *resource unit* could be replaced by *rit*) could be either qubit or classical bit. The total efficiency is mathematically given by

9 $$\begin{aligned} {\mathfrak{E}} = \frac{K}{N} = \frac{K}{Q+M}, \end{aligned}$$

*N* (*N* = *Q* + *M*) being the amount of resources used in a QKD (both bits and qubits), *Q* is the amount of qubits used in a QKD, and *M* is the amount of bits used in a QKD. Taking into account Eq. (8), one obtains

10 $$\begin{aligned} {\mathfrak{E}} = \frac{K}{Q+M} = \frac{R \cdot Q}{Q+M}. \end{aligned}$$

The quantity *M* could be represented as a sum: $\sum _i M_i$, where $M_i$ are the bits announced in the *i*th procedure of a QKD protocol. As can be easily verified by taking a look of a given QKD protocol, the amount of bits announced in a given procedure ($M_i$) depends on the amount of initially transferred qubits (*Q*). Hence the terms $M_i$ could be regarded as functions of *Q* ($M_i$ = *f*(*Q*)), like the key length *K*, i.e., $M_i$ = $m_i$ $\cdot Q$. We thus have

11 $$\begin{aligned} {\mathfrak{E}} = \frac{R \cdot Q}{Q+M} = \frac{R \cdot Q}{Q + \sum _i M_i} = \frac{R \cdot Q}{Q + \sum _i m_i \cdot Q} = \frac{R \cdot Q}{Q + Q\sum _i m_i} = \frac{R}{1 + \sum _i m_i}. \end{aligned}$$

The range of the index *i* and the values of $m_i$ depend on the procedures executed in a QKD protocol, as shown in the Supplementary Material. Proceeding exactly as before (expressing $\mathcal {R}$ in terms of the qubit efficiency *E*), we find that there exists a quantity ${\mathfrak{R}}$ that is expressed by

12 $$\begin{aligned} {\mathfrak{R}} = k \cdot {\mathfrak{E}},\quad [\textit{bits}/\textit{s}] \end{aligned}$$

This quantity is called *total key rate*. The coefficient *k* is given by

13 $$\begin{aligned} k = k_1\cdot c_c + k_2\cdot c_q. \end{aligned}$$

The quantity *k* is the *joint pulse repetition frequency*, $c_c$ is the pulse repetition frequency of the classical source, and $c_q$ = *c* (i.e., pulse repetition frequency of the quantum source). The classical source is the device generating the messages transferred over the public classical channel of QKD. In the above expression, the coefficients $k_1$ and $k_2$ are defined as follows

14 $$\begin{aligned} k_1= & {} \frac{M}{N}, \end{aligned}$$

15 $$\begin{aligned} k_2= & {} \frac{Q}{N}. \end{aligned}$$

Following the above equations, *k* appears to be the average clock rate (pulse repetition frequency) of the QKD protocol. The total key rate takes into account both quantum and classical communication processes of a QKD protocol. In other words, ${\mathfrak{R}}$ represents the actual key rate of a QKD, because the final key (the result of a QKD) is obtained only after both quantum and classical communications (between the parties) are complete.

## Results

We apply the total key rate concept to the original twin-field QKD^9^. Details about determining the $M_i$ ($m_i$) coefficients for the case of TF-QKD are given in the Supplementary Material. Having determined $M_i$ of a given QKD protocol, one is able to compute its total efficiency ${\mathfrak{E}}$ (total key rate ${\mathfrak{R}}$, correspondingly).

**Figure 1:**
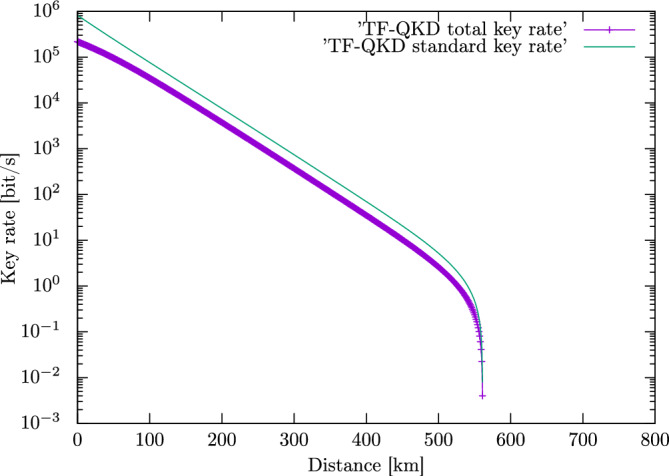
Total key rate ${\mathfrak{R}}$ of the original TF-QKD^9^. We also depict the graph of the key rate given in Ref.^9^ (standard tight line). The pulse repetition frequency of the quantum source $c_q$ is assumed to be equal to the pulse repetition frequency of the classical source $c_c$ and has the value of 1 GHz. We use the settings presented in Ref.^9^ for evaluating the key rates (total key rate and key rate of^9^). These settings involve: detector efficiency, error correction efficiency, dark count probability, pulse intensities and so on.

The result of applying the total key rate to the TF-QKD is depicted in Fig. 1.

## Discussions

In this section we make remarks about: **(1)** the influence of finite-size effects to the efficiency, **(2)** the application of efficiency to QSC protocols, and **(3)** the influence of applying different error correction and privacy amplification algorithms to the efficiency (key rate).

**(1)**. As shown in^13^, the finite size effects affect the value of parameter *R*. Hence, the value of the efficiency changes accordingly. However, the relation between *R* and efficiency (identity $E = R$) still holds. In the case of finite-size QKD, fluctuation corrections are included^13^. Mainly, the corrections are applied to the quantities related to the process of parameter estimation. For example, such a quantity is the probability of (eavesdropping) check basis in an efficient BB84-QKD protocol^13^. In order to overcome the finite-size effects, this probability is chosen so that its value is great for small amount of transferred qubits in a QKD protocol, and its value is small for great amount of transferred qubits in a QKD protocol. As a result of the changes in the QKD protocol parameters, the efficiency varies reciprocally. This means that for higher fluctuation corrections (smaller amount of transferred qubits), the efficiency decreases. The decrease in the efficiency follows from the fact that the percentage of the decoy (check) quantum systems increases in the QKD protocol. The increase of the amount of decoy systems leads to increasing the classical bits exchanged between the parties in a QKD protocol. As shown in Section 3 (Eq. (9)), the efficiency reduces when the amount of classical bits in a protocol increases.

With respect to the evaluation example given in Section 5 (and Supplementary Material), finite-size corrections are necessary for the probability of choosing decoy intensities ($P_{v,w}$, see the Supplementary Material for reference) and the probability of choosing *Z* basis (check basis) [*Note*: The probability of choosing *Z* basis is initially 1/2. However, for extremely small amount of transferred qubits, this probability may need to be increased.].

**(2)**. There exist schemes for directly transferring information (or secrets) in a secure manner. These schemes are known as quantum secure communication (QSC) protocols^14–29^. Note that QSC could be used for distributing cryptographic keys as well. The QSC protocols are divided into two groups: quantum secure direct communication (QSDC)^14–21^ and deterministic secure quantum communication (DSQC)^22–29^. They differ in the way of transferring messages over the communication channel. In the QSDC, the message (key) is transferred only by using a quantum channel. In the DSQC, an auxiliary classical information is required for reading out a message encoded in a quantum system.

In terms of QSC, the equivalent of the parameter *secret key rate* is *secret data rate*. The efficiency of a QSC scheme is determined in a different manner than the efficiency of QKD. The difference is that no information (relevant information) is discarded during QSC^14^. This means that in the case of QSC the parameter *R* (positioned in the numerator of the efficiency expression) needs to be replaced by the overall amount of data transferred by a single QSC protocol run. Another difference between QSC and QKD is probably the number of parameters $M_i$ (or $m_i$) and their values^20,21^. This occurs due to difference between the post-processing phases of QKD and QSC. So, being adapted to the QSC scenario, the efficiency (or total rate) could be used for evaluating and comparing distinct QSC protocols.

**(3)**. We should also mention that error correction and privacy amplification procedures could be evaluated differently. In the Supplementary Material, the error correction (key reconciliation) is evaluated in a general way; no specific algorithm is applied. However, it is possible for one to select and apply certain key reconciliation procedure (e.g., LDPC-based algorithm, Polar-code-based algorithm, CASCADE algorithm, Winnow algorithm, etc.^30,31^). Applying certain algorithm to the proposed efficiency evaluation means picking a given value of the parameter *f* (efficiency of the error correction algorithm). As known, *f* is related to the amount of disclosed bits during the key reconciliation procedure. According to the privacy amplification procedure, as shown in the Supplementary Material, Toeplitz-based algorithm is adopted for calculating the efficiency (total secret key rate) of a TF-QKD. Similar to the case of error correction procedure, one could choose to apply a different privacy amplification algorithm when evaluating the efficiency of a QKD scheme. Note that the expression for determining the bits transferred during privacy amplification is not general. If one decides to use another privacy amplification algorithm, one needs to adopt different expression (way of determining those bits). It is certain that applying different algorithms for both error correction and privacy amplification leads to different $M_i$ (more precisely $M_8$ and $M_9$) values. In this connection, one could infer that different error correction and privacy amplification algorithm leads to distinct efficiency values.

## Conclusion

The article is concerned with defining the total efficiency ${\mathfrak{E}}$ and total key rate ${\mathfrak{R}}$ of a QKD. Also the relation between efficiency and key rate is determined (see Eq. (12)). It is shown that the qubit efficiency *E* (part of the total efficiency) amounts to the standard key rate definition *R* (for instance, the key rate given in Ref.^9^). This equivalence is displayed in Eq. (8). The total efficiency of a QKD is found to be a reduction of the qubit efficiency (see Eq. (11)). Note that this result is compliant with the practical reasoning. The total rate ${\mathfrak{R}}$ is represented as a product between the total efficiency and joint (or overall) pulse repetition frequency *k* of the QKD system (Eq. (12)). The parameter *k* is given as a superposition of both pulse repetition frequency of the quantum source and pulse repetition frequency of the classical source. In this way *k* (correspondingly ${\mathfrak{R}}$) takes into account both quantum and classical parts of a QKD system. Moreover the other part of ${\mathfrak{R}}$, namely the efficiency ${\mathfrak{E}}$, takes into account all the resources (both qubits and bits) used in a QKD protocol. In this regards one could claim that ${\mathfrak{R}}$ is a quantity that completely characterizes a QKD system as opposed to $\mathcal {R}$ (the actual value of the standard key rate; see Eq. (6) for reference). In this regard, ${\mathfrak{R}}$ (or ${\mathfrak{E}}$) could be used for examining existing QKD and QSC schemes or schemes to be developed.

Figure 1 shows the application of ${\mathfrak{R}}$ to the original twin-field quantum key distribution protocol, i.e., the total key rate of TF-QKD is calculated and displayed as a function of the operation distance. The total key rate is compared to that of Ref.^9^ (the quantity $\mathcal {R}$). The curve of the total key rate have lower values with respect to the curve of $\mathcal {R}$ (Ref.^9^). This is a logical result because of the fact that for a given pulse repetition frequency ${\mathfrak{R}}$ involves not only the quantum communication of a QKD (qubits) but also the classical communication (bits). Note that quantum and classical communications are carried out in a sequential order (parties complete the quantum communication and then they perform the classical communication).

As a result, the current work suggests that either the total key rate or the total efficiency needs to be used for characterizing (used for evaluating) QKD protocols. This follows from the fact that both quantities take into account all the resources and all of the procedures in a QKD process.

### Supplementary Information

Supplementary Information.

## Data Availability

All data generated or analysed during this study are included in this published article and its supplementary material.
