# Supplementary material for: On the (relation between) efficiency and secret key rate of QKD
Source: Sci Rep. 2024 Feb 13;14:3638. doi: 10.1038/s41598-024-54246-y (PMC10864355; doi:10.1038/s41598-024-54246-y)
Supplement: Supplementary file 1 — Supplementary Information. [file 41598_2024_54246_MOESM1_ESM.pdf]

Supplementary Material "On the (relation between) efficiency and secret key rate of QKD"

Georgi Bebrov[1*]

[1*]Telecommunications Department, Technical University of Varna, 1 Studentska Street, Varna, 9010, Bulgaria.

Corresponding author(s). E-mail(s): g.bebrov@tu-varna.bg;

In the following lines we determine the values of the quantities M_i as the original twin-field QKD (TF-QKD) is performed.

First we remind the procedures of the original TF-QKD [1]. Note that only the procedures of the post-quantum stage of the protocol are considered (from Procedure 4 to Procedure 7; take a look at the Supplementary Material of Ref. [1]). The reason for this is that M_i have the value of 0 in the procedures prior to Procedure 4, *i.e.*, such procedures are of no importance when the total efficiency is calculated. These procedures of concern are [1]:

4. Charlie interferes the incoming pulses and records which detector clicks. When the quantum communication is over, he publicly announces all the runs where his detector 0 (1) clicked; Alice and Bob will correspondingly set a variable χ equal to 0 (π). All the runs where none of or both of his detectors clicked are discarded.

5. After the previous step is complete, Alice announces the intensities μ_a, the basis phases β_a and the phase slices $\Delta_{k(a)}$ and Bob announces the runs where his values match Alice's. The users discard all the mismatched runs. Then they disclose the bit values of the matched runs except those in basis X and intensity class u, which are kept secret.

6. The users perform a coordinated random permutation of the bits. For the bits in basis X and intensity class u, Bob draws Alice's bit phase α_a from the relation $\chi = |\alpha_b - \alpha_a|$. The users can then distill a key bit equal to 0 (1) when $\alpha_a = 0$ (π). A raw key is formed by concatenating these bits. All the remaining bits, fully disclosed in the previous step, are used to perform the decoy-state

parameter estimation and test the channel against the presence of Eve.
7. The users run classical post-processing procedures such as error correction and privacy amplification to distil the final secret key from the raw key.

For determining the values of M_i, we need only some steps from the above procedures. The steps are the following:
(1) Charlie announces the results of his measurements;
—Users' announcement
(2) Alice announces bases;
(3) Alice announces intensities;
(4) Alice announces phase slices;
(5) Bob announces the matching runs;
(6) Disclosure of key bits produced by WCPs of Z basis and classes u, v, w;
(7) Disclosure of key bits produced by WCPs of X basis and classes v, w;
—Post-processing
(8) Error correction;
(9) Privacy amplification.

Suppose Q number of qubits (weak coherent pulses, WCPs) are transferred by Alice (Bob) in the beginning of the TF-QKD protocol. Then, in step (1) Charlie announces $Q_\mu \cdot Q = M_1$ bits. This value represents the amount of conclusive measurements (clicks) done by Charlie.

During step (2) Alice announces $M_2 = Q_\mu \cdot Q$ bits. She informs Bob about the bases (X or Z basis) of the transferred qubits (being conclusively measured).

In step (3) Alice announces $M_3 = (P_u \cdot n_u + P_v \cdot n_v + P_w \cdot n_w) \cdot Q_\mu \cdot Q$ bits, where P_u, P_v, and P_w represent the probabilities of transferring WCPs of classes u, v, and w, respectively. The quantities n_u, n_v, and n_w represent the sizes of the binary code words used for identifying the classes u, v, and w in the classical communication. Since $P_w = P_v \ll P_u = 1 - P_v - P_w \lesssim 1$ (see Supplementary Material of Ref. [1]) we assume that $M_3 \approx (P_u \cdot n_u) \cdot Q_\mu \cdot Q$. For the case of three intensity classes, one could adopt the following code words: $u \to 0$ ($n_u = 1$); $v \to 10$ ($n_v = 2$); $w \to 11$ ($n_w = 2$). In this case M_3 becomes $M_3 \approx P_u \cdot Q_\mu \cdot Q$.

In step (4) Alice announces $M_4 = m \cdot Q_\mu \cdot Q = 4 \cdot Q_\mu \cdot Q$ bits, where $m = \log_2 M = 4$ and $M = 16$ is the number of phase slices.

In step (5) Bob announces $M_5 = Q_\mu \cdot Q$ bits [one bit per run (measurement): {0,1}, 0 → no match, 1 → match] to inform Alice about the matching runs (runs with identical intensity classes and bases).

After the phase randomization sifting, $\frac{1}{16} Q_\mu \cdot Q$ amount of key bits remain.

After the basis sifting, $\frac{1}{32} Q_\mu \cdot Q$ amount of key bits remain.

In step (6) approximately one half of the key bits are disclosed. This means that $M_6 = \frac{1}{64} Q_\mu \cdot Q$ bits are announced.

In step (7) part of the remaining key bits are disclosed. This part amounts to $M_7 = P_{v,w} \frac{1}{64} Q_\mu \cdot Q$, where $P_{v,w}$ is the probability of transferring WCPs with intensities of both v and w classes. This probability is related to Ω_{unt}, untagged fraction of detection events (zero-photon or single-photon detections)

[1]: $P_{v,w} = 1 - \Omega_{\text{unt}}$.

In step (8) $M_8 = \frac{1}{64} f \cdot Q_\mu \cdot H(E_\mu) \cdot Q$ bits are announced. The quantity M_8 represents the bits disclosed during the error correction (key reconciliation) procedure. The parameter f is the efficiency of the error correction algorithm, E_μ is the quantum bit error rate (QBER) of the pulses with intensity μ, and $H(.)$ is the Shannon entropy.

In step (9) we assume that the Toeplitz-based privacy amplification procedure is performed [2]. Such a privacy amplification procedure is known of announcing $g = x + y - 1$ bits, where x is the length of the error-corrected key and y is the length of the final (secret) key. For the case considered herein, x equals to $\Omega_{\text{unt}} \frac{1}{64} Q_\mu \cdot Q - M_8$, where the first term represents the sifted key length. In the context of the original TF-QKD, the *sifted key* is the binary sequence obtained after both basis and phase randomization sifting procedures. The length of the final key y is equal to $R \cdot Q$, where R is the key rate presented by [1]. Thus $M_9 = g = \Omega_{\text{unt}} \frac{1}{64} Q_\mu \cdot Q - M_8 + R \cdot Q - 1$.

We should note that the coefficient $\frac{1}{64}$ represents the sifting procedures and the parameter estimation procedure: $\frac{1}{64} = \frac{1}{16} \cdot \frac{1}{2} \cdot \frac{1}{2}$, where the first factor reflects the phase randomization sifting, the second factor reflects the basis sifting, and the third factor reflects the parameter estimation coefficient (this coefficient amounts to the fraction of key bits that remains after the parameter estimation procedure). Note that the value of the third factor is approximate (see the paragraphs concerning the expressions of M_6 and M_7).

Based on the analysis in the above lines, we find that M_i are defined as follows (for the case of the original TF-QKD)

$M_1 = Q_\mu \cdot Q;$
$M_2 = Q_\mu \cdot Q;$
$M_3 = P_u \cdot Q_\mu \cdot Q;$
$M_4 = 4 \cdot Q_\mu \cdot Q;$
$M_5 = Q_\mu \cdot Q;$
$M_6 = \frac{1}{64} Q_\mu \cdot Q;$
$M_7 = [1 - \Omega_{\text{unt}}] \frac{1}{64} Q_\mu \cdot Q;$
$M_8 = \frac{1}{64} f \cdot Q_\mu \cdot H(E_\mu) \cdot Q;$
$M_9 = \Omega_{\text{unt}} \frac{1}{64} Q_\mu \cdot Q - M_8 + R \cdot Q - 1.$

The coefficients m_i related to M_i ($M_i = m_i \cdot Q$) have the form

$m_1 = Q_\mu;$
$m_2 = Q_\mu;$
$m_3 = P_u \cdot Q_\mu;$
$m_4 = 4 \cdot Q_\mu;$
$m_5 = Q_\mu;$
$m_6 = \frac{1}{64} Q_\mu;$
$m_7 = [1 - \Omega_{\text{unt}}] \frac{1}{64} Q_\mu;$
$m_8 = \frac{1}{64} f \cdot Q_\mu \cdot H(E_\mu);$
$m_9 = \Omega_{\text{unt}} \frac{1}{64} Q_\mu - m_8 + R - \frac{1}{Q}.$

Using these m_i we obtain the result depicted in Figure 1 of the main paper.

References

[1] Lucamarini, M., Yuan, Z., Dynes, J., Shields, A.: Overcoming the rate–distance limit of quantum key distribution without quantum repeaters. Nature **557**, 400 (2018)

[2] Yuan, Z.L., et al.: 10 mb/s quantum key distribution. J. Light. Technol. **36**, 3427–3433 (2018)
